# Legal regulatory frameworks of radon spas in central Europe

**DOI:** 10.1007/s00411-026-01203-0

**Published:** 2026-02-18

**Authors:** Peter Mihók, Katarína Liptáková

**Affiliations:** Matej Bel University in Banská Bystrica, Banská Bystrica, Slovakia

**Keywords:** Radon, Radon spas, Legislation, Regulatory framework, Radon spa controversy.

## Abstract

This study synthesises publicly available information on legal regulatory frameworks that explicitly or implicitly govern radon spa designation and/or identification in six Central European countries: Austria, the Czech Republic, Germany, Hungary, Poland, and Slovakia. Employing analytical and comparative methods in Social Sciences and Humanities research, this study examines official documents, legal norms, and academic literature to address gaps in understanding radon spa regulation within, and partially beyond, the context of the RadoNorm project. Findings reveal substantial disparities in legally established thresholds distinguishing medicinal and non-medicinal bathing waters based on radon concentrations, ranging from as little as 74 Bq/L in Poland (and potentially only 37 Bq/L in Hungary) to as high as 1,500 Bq/L in the Czech Republic, with intermediate values of 370 Bq/L in Austria and 666 Bq/L in Germany and Slovakia. These pronounced differences present significant challenges for cross-national comparative research and broader conceptualisation efforts concerning radon spa academic definitions. As an incidental finding, this study also identifies varying approaches to workplace safety regulations for spa employees, with Austria and Germany following one approach and the Czech Republic another. Overall, the study contributes to the dissemination of RadoNorm project findings concerning the complexity of the so-called ‘radon spa controversy’, in which radon is framed negatively in public health discourse regarding indoor exposure, yet positively in spa treatment contexts. The findings emphasise the need for interdisciplinary research covering comprehensive medical evidence to understand the rationale behind the divergent regulatory frameworks identified.

## Introduction

The use of radon-rich waters for health “can be traced back to medieval times” (Lettner et al. 2017; see also Becker 2004; Falkenbach 2001; Zdrojewicz and Strzelczyk 2006). Moreover, according to Falkenbach (2001), archaeological findings near the Bad Gastein radon springs in Austria might suggest that radon was among the oldest remedies used in health-related balneotherapy.

Furthermore, Becker (2004) noted that, for millennia, radon therapy “was only indirectly known by its positive health effects” and that, during this period, the terms ‘radium’ and ‘radon’ were used frequently as synonyms. This period ended with the first publications on radon in spa waters, issued by Mache in 1904 and focusing on the Bad Gastein spring. In 1906, the world’s first radon spas were established in Sankt Joachimsthal, now Jáchymov in the Czech Republic. In the same year, a list of radon levels in more than 30 sources across 11 spas in Austria, the Czech Republic, Germany and Italy was published. The quoted author concluded that ‘traditional’ radon spas, such as Bad Gastein and Baden-Baden in Germany, had begun to offer specific radon medical treatments in the early 1910s.

However, in parallel, 20th -century medical research results began to reveal—and later to emphasise—significant negative health impacts of radon. Radon has thus become one of the few naturally occurring elements for which scientific research has reported both positive and negative health impacts (Giacomino and de Michele 2012). Consequently, radon balneotherapy became an increasing concern in radiation protection, starting in the former West Germany in the early 1980s (Sansoni 1994). After the World Health Organisation began presenting exposure to radon as the second leading cause of lung cancer worldwide, second only to tobacco smoke (WHO 2009), public health authorities were compelled to address certain issues surrounding radon balneotherapy within a new risk-benefit framework.

Perceiving radon’s health impacts as enigmatic, Momčilović and Lykken (2005) suggested that prevailing “misconceptions about radon are the result of an overzealous reductionist approach to what essentially is an issue of complexity”. In their opinion, the nature of radon cannot be bootstrapped into a ‘convenient’ simple mental model – instead, it requires a simultaneous, multidimensional view of interactions (as seen in an esoteric mandala). The authors concluded that the reductionistic presentation of the complex human-radon system is bound to look as an optical illusion.

Zdrojewicz and Strzelczyk (2006) appear to be the first academic authors to explicitly use the term ‘controversy’ in relation to empirical existence of radon medical treatments. In line with their conceptualisation, the above-outlined historical context and the RadoNorm project specific needs, the term ‘radon spa controversy’ was conceptualised for its use in RadoNorm as a potential controversy arising from two opposing narratives concerning radon’s health impacts: the negative framing in public health communication regarding indoor radon exposure versus the positive portrayal of radon in spa settings (Himmelbauer et al. 2024). This article presents the results of a research, conducted within and beyond RadoNorm, into the key legal and regulatory frameworks pertinent to this context. A key part of the Discussion section deals with the question of whether existing regulatory frameworks could potentially be related to the ‘dual narrative’ aspect of this controversy, or whether they rather provide a specific context to the framing of the controversy researched within RadoNorm.

Conceived partially as an independent initiative by the authors, this study aims to synthesise publicly available, scientifically valid information on core legal and regulatory frameworks that define or legally influence designation and/or recognition of radon spas in Central Europe. It reports desk research results for six Central European countries (Austria, the Czech Republic, Germany, Hungary, Poland, and Slovakia), which were considered relevant for comparison due to their rather closely shared and historically significant spa tradition. It excludes other forms of radon therapy apart from balneotherapy, specifically inhalation of radon-rich air and ingestion of radon-rich water. This allows for this study to align its focus more closely with the RadoNorm’s finding that European spas traditionally denote facilities providing healthcare services incorporating balneotherapy, contrasting with the broader non-European academic usage of the term ‘spa’ (Mihók and Marčeková 2022).

Additionally, the Results section provides a brief overview of selected official information on measures to protect radon spa employees from potential health risks associated with workplace radon exposure. This information, found only for Austria, the Czech Republic and Germany, was identified incidentally during examination of documents.

Our practical aim is to address particularly a gap in the Social Sciences and Humanities (SSH) discourse regarding the ‘societal context of radon as treatment’ identified in the RadoNorm project (Tomkiv et al. 2021). As experts in the public sector and tourism, and to a smaller extent in political science, we aim to expand upon the pioneering research initiated by RadoNorm into the conflicting ‘cure or carcinogen’ narratives (Geysmans et al. 2022) and the perceptions of these narratives among stakeholders in Austria and Germany, as well as the specific context of their use in the Czech Republic (Himmelbauer et al. 2024). By incorporating Hungary, Poland, and Slovakia, we have sought to establish an initial stand-alone document that could be used to support discussions regarding potential follow-up research to RadoNorm within a broader European context.

## Methods and materials

This study is primarily descriptive—though partially comparative in the Discussion section—and is based entirely on analytical, comparative, and integrative research methods commonly employed in the SSH for the examination of academic and official documents. Specifically, the study involved analysing the content of all materials identified as relevant, followed by comparing and synthesising relevant findings to identify recurring themes and conceptual links.

The materials used to prepare the Results section consist solely of legal norms, academic studies, and official documents (all quoted or paraphrased, and referenced). They were identified through a non-systematic search process: some materials were suggested during consultations with RadoNorm colleagues involved in Subtask 6.4.3 of the RadoNorm project (‘Investigating and addressing risk perception phenomena of radon spas’), while others were found solely through internet-based searches conducted by the corresponding author of this article. Additionally, for Slovakia (the only country not covered by Subtask 6.4.3 of the RadoNorm project), relevant information was obtained by the authors through requests submitted under national freedom of information legislation.

With the exception of Poland, we could not identify English translations of the legally valid documents from which we needed to extract data and information. Consequently, alongside English-language sources, our analysis incorporated legal and official texts in Czech, German (for both Austria and Germany), Hungarian, and Slovak. A range of translation techniques was employed, including AI-assisted verification, with all final texts reviewed by the authors to ensure both necessary accuracy and understandability, as discussed in the ‘Limitations of the Study’ section.

The limited number of materials used in this study was deemed sufficient because they provided all the data and information needed for RadoNorm Subtask 6.4.3 (summarised in the Results section) and were considered relevant and accurate by the subtask’s team. A comprehensive systematic review of all relevant databases to identify other potentially existing literature could not be implemented, as Subtask 6.4.3 was contracted only as a modest initial exploratory investigation with limited capacity for a small research team.

The research presented in this article was conducted exclusively by the authors, thanks to their formal qualifications in tourism and public economics (PM) and public economics and political science (KL). Members of the RadoNorm Subtask 6.4.3 team indicated that, as their expertise lay in other fields, they could not participate directly in this research, and therefore limited their contributions to reviewing the findings related to their respective countries. This split of competence is related not only to the specific qualifications of the Subtask 6.4.3 team members, but also to the limited capacities and mandates contracted for this subtask within RadoNorm, as briefly addressed in the Discussion and also implicitly presented in the subtask final report (Himmelbauer et al. 2024).

## Results

In line with the two above-outlined practical aims, this section summarises the key findings of relevance, structured by country. Brief notes have been appended to the end of each subsection to address research findings of peripheral relevance to our practical aims, or to acknowledge limitations in identifying certain information the relevance of which emerged only following the initial analysis of findings summarised in the Results section.

In this section, inconsistent terminology regarding units of becquerels per litre (Bq/L) has been intentionally maintained when referencing quoted legal and official documents. This approach was adopted with the aim that, should these findings be used by readers in English-language publications of their research, the translated phrases would be as faithful as possible to the original non-English regulatory texts. Furthermore, the umbrella term ‘the threshold value’ has been deliberately applied to refer to these legally relevant values for reasons detailed in the Discussion section.

Inspired by Ritter and Gaisberger (2020), we have proactively incorporated—and, in several instances, independently calculated—values in the legacy unit of nanocuries per litre (nCi/L). This was also needed to address a remark by a Slovak medical expert, cited in the Discussion, which concerns an exact threshold figure adopted in Germany and Slovakia.

### Austria

According to the European Committee of the Regions (2026a), within Austria’s system of concurrent federal powers (or more specifically, its division of competences), central (i.e. federal) authorities have “legislative and executive responsibility for public health including health professions, with the exception of death care, rescue services and the local health service”; regional (*Länder*) authorities are responsible for, among other things, natural healing resources; and local authorities are responsible, among other things, for health administration. In this specific context, according to Ritter and Gaisberger (2020), the [federal] “statutory provisions for the administration of radon [in balneotherapy] require a minimum activity of” 370 Bq/L (10 nCi/L).

Our research revealed that the above-cited requirement is applied in the annexes of the ‘Healing Resources and Health Resorts Acts’ (*Heilvorkommen- und Kuranstaltengesetz*) of the individual federal states (*Länder*). For the sake of terminological clarity and alignment with established academic English (see Mihók and Marčeková 2022), these Acts are hereafter referred to as the Acts on ‘Natural Curative Springs and Spas’. For instance, Annex 1 to the Act on Natural Curative Springs and Spas of [the City-State of] Vienna requires that for bathing treatments, natural springs can be considered as curative if**—**regardless of the amount of dissolved solids**—**the radon content is at least equivalent to 370 Bq/kg.

According to the Austrian Radon Competence Centre (AGES 2025), radon protection regulations in Austria apply uniformly to “mines and other underground work areas, tourist mines and show caves, radon spas, water supply facilities”, and establishments with workplaces in ground floors and basements located in radon protection areas. These establishments must commission authorized monitoring bodies to measure radon concentrations—and if the reference value of 300 Bq/m³ is exceeded, they must also report to the competent provincial authority how they will implement optimization measures to reduce the radon concentration.

Within the RadoNorm project, seven spa facilities were identified as framing radon in English or German language texts (Geysmans et al. 2022). They are located in three spas (spa towns)—Bad Gastein, Bad Hofgastein, and Bad Zell—all of which are members of the radon spa association EURADON (2026).

We have not been able to find any information relevant to a rationale for the significantly different threshold value adopted in Austria (370 Bq/L) compared to the value of 666 Bq/L adopted in Germany and referenced by some internal organisations (see Saman 2000), as also discussed in the Discussion section. For this reason, we chose to cite the German-language article by Ritter and Gaisberger (2020) since it is the only peer-reviewed academic literature we found to address the use of these different values in these two states for the same purpose.

### The Czech Republic

In the Czech Republic, a naturally occurring mineral water can be considered a natural medicinal source thanks to its radon content only if the radon radioactivity exceeds 1,500 Bq/L (which equals approx. 40 nCi/L) (Goliáš et al. 2016, 2022). This requirement is set by the Act [of the Czech Republic] No. 164/2001 Coll. on natural medicinal resources, natural mineral water resources, natural medical spas and spa areas (the Spa Act). In particular, this law grants natural waters exceeding 1,500 Bq/L radon radioactivity the same legal status as other medicinal sources, meaning they can be used for therapeutic purposes (and advertised accordingly) regardless of the natural source (i.e. water, gas, or peloid) or the element(s) to which the healing powers are primarily attributed (chemicals, minerals, radioactivity, etc.).

The medical spa (in Czech ‘Léčebné lázně’) in the town of Jáchymov, a sole Czech member of EURADON, is the only spa in the Czech Republic the bathing waters of which meet the aforementioned legal requirement for offering/advertising radon medical treatments. This spa declares its compliance with all radon safety-related requirements through a Certificate held from the Czech State Office for Nuclear Safety, emphasizing that this certificate concerns the safety of both patients and spa employees (Léčebné lázně Jáchymov 2025).

As with the Austrian case, we have sought to identify a rationale for adopting a threshold value that differs from the German equivalent and, moreover, is distinctly higher. But despite our efforts, the justification for setting the threshold at exactly 1,500 Bq/L remains unclear to us.

### Germany

According to the European Committee of the Regions (2026b), within Germany’s system of concurrent federal powers, health policy implementation in Germany “is decentralised and differs strongly between the areas”. Implicitly according to this resource, this is the reason why there also exist specific intermediate level (“Kreise”) authorities, i.e. apart from relevant central (i.e. federal) and regional (Länder) authorities. While central authorities, among other things, set the general framework for the organisation and performance of the health sector, regional (Länder) authorities are responsible, among other things, for legislation on ‘medical corps’ (‘Gesundheitsdienst’) (note: in our opinion, the term ‘medical corps’, explicitly present in the quoted English language source, most probably refers to ’Public Health Service units’ rather than ‘military medical units’).

In the above-outlined specific context, the federally valid regulatory framework relevant to our research is set by the [quality standards] document titled “Begriffsbestimmungen / Qualitätsstandards für Heilbäder und Kurorte, Luftkurorte, Erholungsorte” (in English, “Definitions/Quality Standards for Spas and Health Resorts, Air-Health Resorts, Recreation Resorts”). At present, it is being published jointly by the Deutscher Heilbäderverband e.V. (German Health Resorts Association) and the Deutscher Tourismusverband e.V. (DTV, German Tourism Association). According to the German Health Resorts Association, as a “substantive component of the spa laws and ordinances of the [individual] federal states” (Länder), this document sets “generally recognised principles of the spa and health resort sector” (Deutscher Heilbäderverband e.V. 2026a). Among others, it sets rules based on which natural waters used for bathing can be considered natural healing waters based on their chemical composition or their physical properties, according to the scientific principles.

As of the finalisation of this article, the most recent, 14th edition of this document was adopted on 4 November 2023 (Deutscher Heilbäderverband e.V. 2026b). It stipulates that “radon-containing waters” (radonhaltige Wässer) can be considered natural healing waters when the value of their radon content reaches “the minimum value” of 666 Bq/L (18 nCi/L) (Deutscher Heilbäderverband e.V. & DTV, 2023). Furthermore, it states that the use of radon-rich waters is considered medicinal only in relation to bathing; i.e. in contrast to carbon dioxide-rich waters, for which this document sets different thresholds for bathing therapies and drinking therapies.

In order to gain a basic understanding of how the aforementioned quality standards document is transposed into regional-level legal regulatory frameworks, we reviewed the relevant ordinances from the two federal states (Länder), Bavaria and Saxony, deliberately selected by us due to the presence of radon spas. In both instances, our review indicates that these instruments—the full titles of which are provided in the References—incorporate the quality standards by reference, effectively mandating the 666 Bq/L threshold as a legal prerequisite for radon-rich bathing waters to be classified as curative.

The Federal Office for Radiation Protection (Bundesamt für Strahlenschutz, BfS), which sets guidelines and regulations for radon exposure in workplaces and public areas, states that “radon applications are not advised as spa treatments” (BfS 2025a). Similarly to Austria, radon spas are explicitly listed among the types of businesses that are legally obliged to measure radon concentrations at workplaces in accordance with the Radiation Protection Act (BfS 2025b). “Since employees at workplaces in radon spas and radon healing resorts are exposed to radon more persistently than patients, their exposure to radon is regularly checked and registered in order to protect them from radon at work” (BfS 2025a).

Nine spa facilities at/near these eight towns have been identified within RadoNorm to advertise radon medical therapy in English or German (in alphabetical order): Bad Brambach, Bad Kreuznach, Bad Schlema, Bad Schmiedeberg, Bad Steben, St. Blasien (note: the facility identified in RadoNorm, located in the nearby village of Menzenschwand, was closed at the end of 2025), Neualbenreuth, and Weissenstadt. Apart from them, EURADON (2026) also lists the spa resort (Kurbad) of the town of Altenberg among its German members.

### Hungary

According to Bender (2003), as quoted by Szabó et al. (2023), “in Europe, Hungary has the strictest process for declaring medicinal water”. With regard to this process, we identified the Decree of the [Hungarian] Ministry of Health on natural healing factors (No. 74/1999 (XII. 25.) as the key regulatory legal instrument. Of particular relevance is Appendix 2 (Part I, Sect. 1(e)(ea) of this Decree, which lists seven naturally occurring constituents in bathing waters that are deemed potential natural healing factors (i.e. factors of physical, chemical, or radiological origin). For each of these seven constituents, the Decree prescribes a minimum concentration value that must be met for the water to be considered potentially therapeutic for bathing use on the basis of the presence of the respective constituent. For radon, as one such potential constituent, the Decree sets a minimum radon activity concentration of 37 Bq/L (1 nCi/L) as a necessary condition for classifying natural water as suitable for curative bathing use. At the same time, the Decree contains a separate provision under § 19 stipulating that the presence of one or more of these seven natural constituents may be linked to a therapeutic effect of bathing in such waters only if the presumed therapeutic effect associated with the respective constituent(s) is supported by properly documented and evaluated medical research carried out in accordance with scientifically recognised methods (see also Somlai et al. 2007).

In the above-outlined specific Hungarian legal context, according to Nagy et al. (2008), “radon therapy is defined as a medicinal therapy where the active substance is known, however neither the dose for curative effect nor the duration of required treatment is identified” (see also Shahrokhi et al. 2016; Somlai et al. 2007; Szabó et al. 2023).

According to academic literature we reviewed, the Turkish bath facility (fürdő) at Eger is the only spa facility in Hungary which is “founding the spa treatment on the radon content of the water i.e. where radon therapy is performed” (Nagy et al. 2008; see also Shahrokhi et al. 2016).

The list of EURADON’s (2026) member radon spas does not include any located in Hungary. The implicit finding that the radon content at the only Hungarian facility where radon therapy is performed (the Turkish bath facility (fürdő) in Eger) may be lower than the radon content at other spa facilitiy/es in Hungary, as noted by Nagy et al. (2008), is discussed in the Discussion section.

### Poland

In Poland, radon-rich waters can be recognized as medicinal and thus used for medical therapies if the radon content exceeds 74 Bq/L (and if they meet both extraction and hygienic requirements) (Kuciel-Lewandowska et al. 2020; Przylibski et al. 2022). The Polish Geological and Mining Law provides waters with such radon content the same legal status as all other types of “waters of curative properties”, defined by this Law as “underground waters containing no chemical or microbiological pollutants and having naturally changeable physical and chemical properties” (Act [of Poland] of 9 June 2011, Article 5).

Apart from the Świeradów-Zdrój, which is the only Polish member of the European radon spa association EURADON (2026), Lądek-Zdrój is referred in peer-reviewed literature as a spa the bathing waters of which fulfil the above-referred criteria (Nowak and Nguyen Dinh 2017; Przylibski and Żebrowski 1999). According to Grzywa-Celińska et al. (2020), several other Polish spas also utilize radon-rich waters, namely Jedlina-Zdrój, Przerzeczyn-Zdrój, Szczawno-Zdrój, Długopole-Zdrój, and Duszniki-Zdrój. Like all other spas in Poland, those using radon-rich waters that meet the above-quoted legal criteria are required to assess employee and patient radiation exposure under the Polish Atomic Law (Nowak and Nguyen Dinh 2017).

We have not been able to find any information relevant to the rationale for adopting the above-referred threshold at the value of 74 Bq/L, i.e. significantly lower than in Austria (370 Bq/L), Czechia (1,500 Bq/L) and Germany (666 Bq/L).

### Slovakia

Slovakia’s Act on Natural Healing Waters, Natural Medical Spas, Spa Localities and Natural Mineral Waters does not contain any reference to radon-rich waters or to radon as such. According to the Regulation No. 100/2006 Coll. of the Ministry of Health laying down the requirements for natural medicinal and mineral waters, medicinal waters can be recognized and classified as “radon waters” if their radon activity exceeds 666 Bq/L (Ministry of Health [of the Slovak Republic] 2023).

According to the information obtained from the Ministry of Health (2023), regular monitoring of legally recognized medicinal resources at spa facilities, which includes measurements of radon content in bathing waters, is conducted once every five years. The Ministry also stated that the most recent measurements implemented in the above-referred context identified the highest radon content in spa bathing waters at 145 Bq/L at the Sklené Teplice spa. Previously, a year-long investigation of radon content in 23 selected thermal water sources at seven selected Slovak spas, plus one frequently visited wellness facility by Blahušiak et al. (2017), found the highest radon content measured to be 435 ± 20 Bq/L particularly at the same spa.

Despite efforts, we were unable to identify any information relevant to the finding that Slovakia adopted the above-referred threshold value identical to the value in Germany, i.e. 666 Bq/L. The only relevant source identified during our desk research in this regard—a statement by the Slovak balneotherapy expert Doležal (2021)—is examined in the Discussion section.

## Discussion

This section discusses potential interpretations of our findings within the scope of our disciplinary expertise (as specified in the Methods and Materials section). Furthermore, it respects the position that RadoNorm “could not and did not evaluate the actual effectiveness and/or safety of radon treatments” (Himmelbauer et al. 2024). Consequently, our analysis does not extend to the natural or medical sciences, except where references to medical research are an inevitable part of the interdisciplinary approach required to discuss research gaps identified by our study.

Given the limitations referred to above, we do not discuss our finding that the examined regulatory frameworks categorise radon-containing waters into two distinct legal groups: medicinal and non-medicinal. However, we consider it pertinent to highlight that, in five of the six countries studied, this division is based solely on one criterion: whether the radon activity concentration (measured in Bq/L) reaches certain minimum values. Consequently, for the purposes of this discussion, we therefore treat these values as thresholds.

Although the umbrella term ‘threshold value’ does not appear in the reviewed documents, we use it in the text below to emphasise the regulatory function performed by these minimum values within the respective legal frameworks. In this regard, we must emphasise that the contexts in which these threshold values were adopted by lawmakers may vary significantly. Therefore, Table 1 also summarises the different types of documents in which these values appear. It is deliberately placed in the Discussion section to avoid any potentially misleading impression that we perceive these threshold values as suitable for scientific comparison in SSH.

While all values in Bq/L listed in Table 1 are incorporated into or referenced within legal norms and/or official documents, we found no references to medical research in these documents, with the sole exception of Hungary. With regard to this de facto unique finding for Hungary, we consider it worth noting that we did not identify any link between the clause referring to medical research (translated in the Results section) and the adoption of the 37 Bq/L value. As implicitly mentioned in the second paragraph, the only remark we can make about this particular value is that it is significantly lower than the values used in similar contexts for similar purposes in Austria (370 Bq/L), Czechia (1,500 Bq/L), and Germany (666 Bq/L).

**Table 1:** Summary of key information identified in legal regulatory frameworks analysis. The radon threshold is used as basis for categorising bathing waters as non-medicinal and medicinal

State	Type of Legislative Instrument	Minimum Radon Threshold	Notes
Austria	Annexes to sub-state-wide Acts	370 Bq/L (= 10 nCi/L)	The threshold is established in attachments/appendices to the laws of individual federal states (Länder) on healing resources and spas or health resorts.
Czech Republic	State-wide Act	1,500 Bq/L (≈ 40 nCi/L)	Act on natural medicinal resources (the Spa Act, No. 164/2001 Coll.).
Germany	Official document referenced in sub-state-wide Acts	666 Bq/L (= 18 nCi/L)	The document titled “Quality standards” (DTV, and German Health Resorts Association, referenced by federal state (Länder) legislation.
Hungary	State-wide Decree	37 Bq/L plus an additional condition	Decree of the Ministry of Health (No. 74/1999). The additional condition is that radon must be confirmed as the key medicinal agent through scientifically recognised research methods and approved by the relevant authority.
Poland	State-wide Act	74 Bq/L	Geological and Mining Act (Act of 9 June 2011).
Slovakia	State-wide Regulation	666 Bq/L	Regulation of the Ministry of Health (No. 100/2006 Coll.)

Sources of data: documents quoted or paraphrased in the Results section (and listed in References)

### Extending the radonorm perspective on radon spa controversy

The core part of RadoNorm research concerned how framings of radon in radon spas’ internet marketing activities (Geysmans et al. 2022) and related perceptions of radon spa employees and other relevant stakeholders contrast with the radon-related awareness public campaigns of health authorities; and the related potential influence on varied radon-risk related perceptions of the general public in Austria, the Czech Republic and Germany (Himmelbauer et al. 2024). In our view, some of our research results extend these RadoNorm findings because they point to contradictions independent of radon spas’ marketing, since they concern solely institutions directly linked to preparations and/or approvals of legal regulatory frameworks.

Perhaps the most notable of such findings concerns the German Health Resorts Association and the German Tourism Association (DTV), referred to in the Results section, which publish a federally valid document (Deutscher Heilbäderverband e.V. and DTV 2023) that we identified as being referenced in sub-state-wide (Länder) Acts. The key reason for this focus is that the relevant radon-related content in this document claims beneficial medical effects for bathing in water with a radon concentration of at least 666 Bq/L, which potentially contradicts the position of the federal authority for radiation protection BfS (2025a), which states that “radon applications are not advised as spa treatments”. This potential contradiction raises questions beyond our current research capacity: by whom, why, and when were these two industry associations mandated to prepare—or at least shape—and publish a document so central to the German legal regulatory framework relevant for RadoNorm. From an SSH perspective, the follow-up question arises as to whom these two German associations are accountable to, and how. We find this relevant also because one or both these associations may have radon spas amongst their members. In this regard, it is worth noting that in all other countries covered in our research, legal regulatory frameworks are set by Acts, or a Decree or Regulation, i.e. by such legal acts that are both prepared and approved by public sector entities accountable to the public.

Potential controversies regarding accountability of some legal entities involved in shaping and/or implementing legal regulatory frameworks pertaining to radon spas may extend the RadoNorm perspective on radon spa controversy at both national and international levels. At both levels, this can be perceived through hypothetical, but not unrealistic, scenarios of public health authorities targeting some relevant RadoNorm findings. In our view, this can perhaps be discussed using the RadoNorm finding by Geysmans et al. (2022) that several radon spas present radon’s health impacts using a “Fountain of Youth” framing.

On a German national level, our findings may go beyond questions concerning the accountability of the two associations referred to above. In our view, further challenges may arise from the fact that “health policy implementation is decentralised and differs strongly between the areas” (European Committee of the Regions 2026b), with related legal and implementation mandates given to all relevant ‘intermediate level’ (‘Kreise’) legal entities. This is because such a complex and specific legal and regulatory context also inevitably influences accountability of legal bodies empowered to decide whether radon spas should be permitted to present radon using a “Fountain of Youth” framing, or perhaps be sanctioned for this practice instead. While our findings do not provide information that could be used to discuss this, they extend the RadoNorm perspective on radon spa controversy by outlining how complex and challenging research into the accountability of legal bodies empowered to evaluate radon spas’ marketing can be.

Furthermore, some of our research results outline that even more challenging questions would arise should controversial radon spas’ marketing be targeted by a joint response from Austrian and German public health authorities. While the RadoNorm report by Himmelbauer et al. (2024) provides health authorities, particularly from these two countries, with a significant amount of primary research results, it does not mention that concurrent powers in these two federal states appear to be fragmented rather differently, as implicitly outlined by our research results. To avoid repetitions, we discuss this specific theme below in the Research gaps subsection.

## Challenges in cross-national comparisons

In the RadoNorm project, primary research was conducted in Austria and Germany using semi-structured interviews. These two countries were chosen after Geysmans et al. (2022) identified as many as nine radon spas in Germany and seven in Austria, while identifying no more than two in any of the remaining EU member states. The respective RadoNorm final report by Himmelbauer et al. (2024) presents the results of these interviews without a comparison among these two countries, and without an explicit justification for why such comparative analysis was omitted. In this sub-section, we therefore discuss certain secondary findings that are relevant in this regard.

While the RadoNorm report explicitly discusses the differing threshold values for the same or very similar regulatory purpose between Austria (370 Bq/L) and Germany (666 Bq/L), its aim and focus did not require a discussion of the potentially relevant context that transcends these two countries in this regard. Therefore, we find it relevant to mention that Saman (2000) linked the threshold value of 666 Bq/L also to various international classifications, namely “the Classification of the International Society of Medical Hydrology, the Société Internationale de Technique Hydrothermale (SITH), [and] The International Association of Spas, Health Resorts and Balneotherapy”. However, in this regard, we have not been able to find any other peer-reviewed academic literature relevant for hypothesising about this context, for instance in relation to the adoption of the same value in Slovakia. Furthermore, we noted that a relevant Slovak expert in balneotherapy commented on this value of 666 Bq/L by asking “could this be a reference to the Revelation of the Apostle John, Chap. 13, verse 1 and verse 18?” (Doležal 2021). We therefore conclude that our research did not yield sufficient evidence to explain the discrepancy between the Austrian (370 Bq/L) and German (666 Bq/L) threshold values. Nevertheless, we considered it relevant to report the above-summarised secondary findings because they may serve as an impetus for future research into the historical, institutional, and cultural contexts of adopting radon-spa-related threshold values internationally.

Additionally, we consider it worth noting that we incidentally identified potentially varying approaches to workplace safety regulations for spa employees. In particular, our research results revealed that while radon-in-air-content measurements in Austria and Germany are legally required to be commissioned by spa operators, the sole Czech radon spa demonstrates compliance with radon-related employee safety requirements through a certificate issued by the Czech State Office for Nuclear Safety. We discuss these findings here because they are, in our opinion, relevant to not conducting interviews in the Czech Republic within RadoNorm’s primary research. We would also like to note that we did not identify any information that would concern radon spa employee protection in Hungary or Poland in any of the material reviewed in this study.

### Challenges with broader conceptualisation of radon spas in central Europe

Across the six Central European countries examined, our findings show even greater differences in the legally established thresholds for radon concentrations in bathing waters than those identified in Austria and Germany (as discussed above). In particular, for the same threshold parameter, Poland has adopted a value as low as 74 Bq/L, whereas the Czech Republic has set it as high as 1,500 Bq/L. Moreover, in Hungary, the lowest of the threshold values we identified, particularly at 37 Bq/L, may potentially be sufficient in this regard within a specific context, as discussed above and below.

As already implicitly noted, particularly the regulatory framework in Hungary may present a very significant challenge in broader conceptualisation efforts concerning unification of radon spas’ definition/determination. In the relevant documents reviewed, we found consensus that Eger is the sole Hungarian spa permitted by the Hungarian regulatory framework to advertise medical effects of its bathing waters based on radon content. In fact, the official tourism promotion agency of Eger links the average radon content of the medicinal spring at Eger, reported as 80 Bq/L (Nagy et al. 2009), with the slogan “even the elderly look youngish” (Visit Eger, 2024). In doing so, the tourism promotion agency applies the ‘[radon as a] Fountain of Youth’ frame identified by Geysmans et al. (2022). By contrast, although the Gellért spa facility in Budapest uses a spring with a much higher radon content, measured at approximately 1,065 ± 35 Bq/L (Kovács-Bodor et al. 2019), we were unable to identify any use of radon-related framing on the spa’s website or in its associated marketing materials.

Potentially, some of our findings might further contribute to terminology and conceptualisation issues concerning the term ‘spa’ identified in research by Mihók and Marčeková (2022). This research identified fundamental differences between European and non-European understandings of the term ‘spa’, with European perspectives emphasising balneotherapy as a more or less compulsory component.

## Limitations of the study

In contrast with the EU-wide scope dealt with in RadoNorm by Geysmans et al. (2022), the geographical scope of our study is limited to Central Europe. In our view, this limit proved sufficient to initially discuss key challenges with how differences in national approaches to establishing legal regulatory frameworks make it very difficult to generalise the broader concept of radon spas. However, the geographical limitation of our study needs to be emphasised, for instance because non-academic literature, such as the self-published book by Liechti (2020), explicitly suggests that national approaches outside of Central Europe may in this particular regard be very different in some countries of the so-called old EU, namely in France, Belgium, or Italy.

We must also acknowledge that certain limitations of our study arise from our inability to consult foreign legal experts both within and outside RadoNorm, which is particularly pertinent given the concurrent legislative frameworks in Austria and Germany. To address this constraint, we implemented two methodological adjustments in the Results section. First, we endeavoured to render relevant legal texts into accessible English, recognising that literal translations might present comprehension challenges for readers without legal expertise, whilst ensuring that all substantive legal content remained accurately conveyed for the purposes of our study. Second, wherever feasible, we sought to validate these translations through citations from peer-reviewed English-language literature. However, as discussed in the sub-section on Germany, we identified a potentially problematic translation in the European Committee of the Regions’ (2026b) rendering of “Gesundheitsdienst” as “medical corps”. We suggest that this German term more appropriately refers to ‘[Public] Health Service units’ rather than military medical units. In our view, this observation underscores the broader methodological challenges inherent in producing scientifically rigorous English translations of legal texts and their subsequent interpretation, both within and beyond the scope of our study.

In the context of limitations discussed above, we emphasise that our study primarily aims to inform post-RadoNorm discussions regarding follow-up research on Himmelbauer et al. (2024), and should therefore be understood as a contribution to discussions about research gaps rather than a definitive source for legal translations and interpretations.

### Research gaps

In our view, combined findings by Geysmans et al. (2022), Himmelbauer et al. (2024) and our study could be followed up by SSH research focused on clarification of institutional roles of relevant actors. Moreover, this may concern not only the actors already identified, but also other potentially relevant actors and stakeholders. Some of our results implicitly suggest that legal bodies endowed with legal rights to evaluate radon spas’ marketing activities, inclusive of a right to impose fines, could be new actors of relevance in follow-up SSH research. This particularly regards those marketing activities that use such frames identified by Geysmans et al. (2022) that might undermine the radon‑related messaging of public health authorities.

However, as already noted, we believe that significant challenges could arise if the follow-up SSH research discussed above were to exclude relevant legal experts. These challenges are likely to be significant particularly in the context of fragmented concurrent powers in Austria and Germany. Due to a lack of opportunity to consult relevant legal experts—consultations not mandated within either RadoNorm or our capacity for this study—we were unable to distinguish between the questions which arose during our research that could potentially be answered via such consultations, and questions necessitating further scientific SSH research.

Similarly, we cannot draw a comparable distinction regarding our findings on the divergent threshold values found in various legal frameworks (see Table 1), as discussed in the subsection ‘Challenges in cross-national comparisons’. To build upon that discussion, we would welcome any research into the extent to which the disparities we identified might relate to the Linear Non-Threshold (LNT) model — which appears to underpin several radon-related public health campaigns — and/or to the ‘ALARA’ (As Low As Reasonably Achievable) principle. While these matters fall outside our SSH expertise, we suspect that some policymakers may prioritise economic feasibility, thus favouring a more lenient regulatory approach for radon spas based on ALARA; while others might choose to emphasise the precautionary principle implicit in the LNT hypothesis, leading to stricter regulatory frameworks or even political bans of radon spas (see, for example, a self-published book by Liechti (2020). In this context, we suggest that SSH researchers could benefit from clarification of the following assumption: while understanding the differences in the radon-spa-related regulatory approaches is primarily a political or legislative matter, assessing their potential impact on health should remain a strictly scientific question, likely falling entirely within the domain of medical science.

Based on the discussion above, we assume that political drivers could also potentially be relevant with regard to our findings that concern empirically existing discrepancies in radon concentration thresholds summarised in Table 1. In this context, we would like to repeatedly emphasise that in the geographically compact region of six EU member states with significantly shared historical context particularly with regard to spas, the discrepancies we identified are very significant. But while we also acknowledge that “the [SSH] empirical research on [political] lobbying has progressed substantially” (Figueiredo and Richter 2014), we intentionally refrain from any more commentaries on suggested future research to explain the above-referred significant deviations. The key reason is that this research gap is, in our view, largely outside our limited qualification. We assume this, as valid interpretation of these deviations seems to inevitably require scientifically valid analysis of medical studies that claim positive health impacts of radon balneotherapy, some of which were noted and mentioned by Geysmans et al. (2022).

The RadoNorm project has, in a sense, reformulated the controversy researched from the ‘radon treatment [controversy]’ (Zdrojewicz and Strzelczyk 2006) to the ‘radon spa controversy’. In this context, we have identified research gaps pertaining to academic use of the term ‘spa’ and some related terms. Only thanks to research needs and initial findings of RadoNorm, it was possible for Mihók (note: the corresponding author of this article) and Marčeková (2022) to prepare a separate study combining project-related and freelance research which is potentially the first piece with this particular focus. The findings presented in this article expand research gaps implicitly discussed by Mihók and Marčeková (2022) particularly concerning cross-border medical tourism. However, our initial outline of substantial regulatory differences provides in our view only an incomplete initial knowledge base necessary for experts in law and tourism to formulate specific research needs in this regard.

In our view, a holistic analysis of the combined findings of Geysmans et al. (2022), Himmelbauer et al. (2024), and our own study reveals significant gaps in interdisciplinary research within this domain. As experts in the public sector, we would particularly welcome research addressing the public interest objectives implicitly discussed by Himmelbauer et al. (2024). In this context, we note that we are unaware of an existence of a comprehensive, up-to-date summary of the radon-spa related legal rights and responsibilities of national health and radiation protection authorities, alongside key actors in the tourism sector — including not only ministries, but also relevant umbrella organisations within medical and/or spa tourism. Given that radon medical treatments are available only in a limited number of EU Member States (see, for example, Liechti 2020), we assume that such a summary is a necessary prerequisite for any discussion on cross-border radon-spa tourism in this context. However, as previously noted, precisely identifying further research gaps in this specialised field might inevitably require the involvement of experts from, among others, the medical and legal sciences.

## Concluding remarks

As SSH researchers from a country without a radon spa, we perceive the final report of RadoNorm subtask 6.4.3 by Himmelbauer et al. (2024) as a response by the project’s SSH research community to several developments. First, it responds to the initiation of academic debate on the medical controversy surrounding radon treatment by medical scientists Zdrojewicz and Strzelczyk (2006). Second, it engages with international empirical evidence summarised—albeit, in our view, controversially—in a self-published book by Liechti (2020). Third, it addresses the scarcity of directly relevant peer-reviewed SSH publications by EU-based authors identified within RadoNorm (Tomkiv et al. 2021).

Within the above-outlined context, RadoNorm SSH research on the radon spa controversy (conducted primarily in 2022–2024) represented the first coordinated effort within the EURATOM ‘Horizon’ research programme to analyse and summarise relevant radon spas’ internet marketing practices (Geysmans et al. 2022), interview relevant stakeholders, and establish an initial SSH knowledge base in this domain (Himmelbauer et al. 2024; see also Tomkiv et al. 2021).

Following RadoNorm’s contractual requirements, this article refrains from reviewing or commenting on medical research on the effectiveness and/or safety of radon spa medical treatments, as acknowledged by Himmelbauer et al. (2024). Maintaining a neutral position towards the radon spas’ empirical existence, our findings complement and/or discuss RadoNorm’s pioneering SSH research results through the identification and examination of the most relevant legal regulatory frameworks concerning radon spas in Central Europe (from the perspective of their study in RadoNorm). We selected six countries forming a compact region with shared spa traditions (in a sense, approximately 75% of facilities identified by Geysmans et al. (2022) as advertising radon medical therapies on their websites fall within our research scope).

In our view, our findings summarised in this article complement and validate the decision to conduct RadoNorm primary research exclusively in Austria and Germany. Independently of RadoNorm research, our findings suggest that regulatory environments relevant to radon spa marketing activities might be very complex specifically in these two countries, partially as a consequence of potentially differing fragmented concurrent powers within these two federations (note: all remaining countries in our research have unitary political systems).

Our study might potentially be the first SSH publication providing results relevant to potential discussions concerning the harmonisation of radon-spa policies across the six countries examined. Our findings and subsequent discussion outcomes suggest that such discussions would require sophisticated legal analysis to navigate potentially overlapping competences in, among others, healthcare, tourism, radiation protection, and consumer rights for legal and legitimate advertising.

Furthermore, the substantial variation in threshold values across the six Central European states covered in our research—from Poland’s 74 Bq/L (respectively Hungary’s 37 Bq/L) to the Czech Republic’s 1,500 Bq/L—underscores a considerable divergence in national regulatory approaches. In the Discussion section, we outline the potential challenges for future research regarding this divergence.

While RadoNorm enhanced our understanding of the radon spa controversy from health-risk perception and ‘dual narratives’ perspectives, understanding why lawmakers adopted such divergent threshold values—as identified and summarized by us in the Table 1—remains challenging for us. In our view, addressing research gaps related to this challenge inevitably requires medical science expertise whose involvement, as mentioned, could not be achieved within RadoNorm for contractual reasons.

## Data Availability

No datasets were generated or analysed during the current study.

## Abbreviations

AGES: Austrian Radon Competence Centre
BfS: [German]Federal Office for Radiation Protection (Bundesamt für Strahlenschutz)
Bq/kg: Becquerels per kilogram
Bq/L: Becquerels per litre
Bq/m³: Becquerels per cubic metre
DTV: German Tourism Association (Deutscher Tourismusverband)
EU: European Union
EURADON: European Radon Spa Association
nCi/L: Nanocuries per litre
SITH: Société Internationale de Technique Hydrothermale
SSH: Social Sciences and Humanities
WHO: World Health Organisation
